# Case Report of Concomitant Diagnosis of Locally Advanced Intrahepatic Cholangiocarcinoma and Solitary Plasmacytoma of T11 Vertebra: Impact on Diagnostic and Clinical Management

**DOI:** 10.3390/curroncol31090382

**Published:** 2024-09-02

**Authors:** Yann Touchefeu, Matthieu Barbaud, Laura Prin-Felix, Edouard Samarut, Bastien Jamet, Luc Ollivier, Damien Bouda

**Affiliations:** 1 Inserm CIC 1413, Hépato-Gastroentérologie, Institut des Maladies de l’Appareil Digestif (IMAD), CHU Nantes, Université de Nantes, F-44000 Nantes, France; 2 CRCI2NA, INSERM UMR1307, CNRS-ERL6075, Université de Nantes, F-44000 Nantes, France; 3 CRCI2NA, INSERM UMR1307, CNRS-ERL6075, Médecine Nucléaire, CHU Nantes, Université de Nantes, F-44000 Nantes, France; matthieu.barbaud@chu-nantes.fr (M.B.); bastien.jamet@chu-nantes.fr (B.J.); 4 Service Hématologie, Nantes University Hospital, F-44000 Nantes, France; laura.prinfelix@chu-nantes.fr; 5 Neurotraumatology and Neurosurgery Department, Nantes University Hospital, F-44000 Nantes, France; edouard.samarut@chu-nantes.fr; 6 Department of Radiation Oncology, Institut de Cancérologie de L’Ouest (ICO), F-44800 Saint-Herblain, France; luc.ollivier@ico.unicancer.fr; 7 IRIS GRIM-Site de Saint-Herblain-Santé Atlantique, F-44800 Saint-Herblain, France; d.bouda@iris-grim.fr

**Keywords:** solitary bone plasmacytoma, cholangiocarcinoma, radiotherapy, immunotherapy

## Abstract

A solitary bone plasmacytoma is a rare tumor. Intrahepatic cholangiocarcinoma is the second most common primary liver cancer after hepatocellular carcinoma. We present the case of a 48-year-old female patient who consulted for recent back pain, with a final diagnosis of T10 solitary plasmacytoma and synchronous intrahepatic cholangiocarcinoma. Imaging suggested cholangiocarcinoma with bone metastasis. The patient underwent neurosurgical management with laminectomy, arthrodesis, and arthrectomy, with biopsies revealing monotypic kappa plasmacytic proliferation. Liver biopsies revealed an adenocarcinoma with expression of cytokeratin 19, cytokeratin 7, N-cadherin, and high expression of carbonic anydrase IX. The plasmacytoma was treated with external radiotherapy. The cholangiocarcinoma was treated with selective internal radiation therapy and concomitant systemic treatment with combinations of cisplatin and durvalumab, with capecitabine during radiotherapy, switched for gemcitabine after completion of irradiation. One year after initial management, imaging revealed a partial metabolic response of the intrahepatic cholangiocarcinoma, and a complete metabolic response of the plasmacytoma. This case illustrates the importance of not ignoring two primary tumors and the management of two concomitant treatments exploiting potential therapeutic synergies and limiting expected toxicities.

## 1. Introduction

This case report outlines the presentation and management of a 48-year-old female patient who initially sought medical attention for dorsal pain. Despite a lack of significant medical history, the diagnostic journey revealed a challenging combination of pathologies—solitary bone plasmacytoma and synchronous unresectable intrahepatic cholangiocarcinoma. The report provides an account of the clinical presentation, radiological characteristics, and the management of therapies aiming at limiting toxicities and exploiting synergistic effects.

## 2. Case Presentation

A 48-year-old female patient presented with back pain. She had no previous history apart from a personal history of colonic polyps (last colonoscopy 5 years ago) and a second-degree family history of colorectal cancer. She was not taking any medication, and had no chronic alcohol abuse. The medical history began with a fall and persistent back pain. The WHO score was 0, and appetite was preserved without any weight loss. There was no neurological deficit, no pain on spinal percussion, and no abdominal pain. Physical examination was unremarkable. Routine laboratory tests were normal, except Gamma glutamyl transferase being three times the upper limit value.

**Figure 1:**
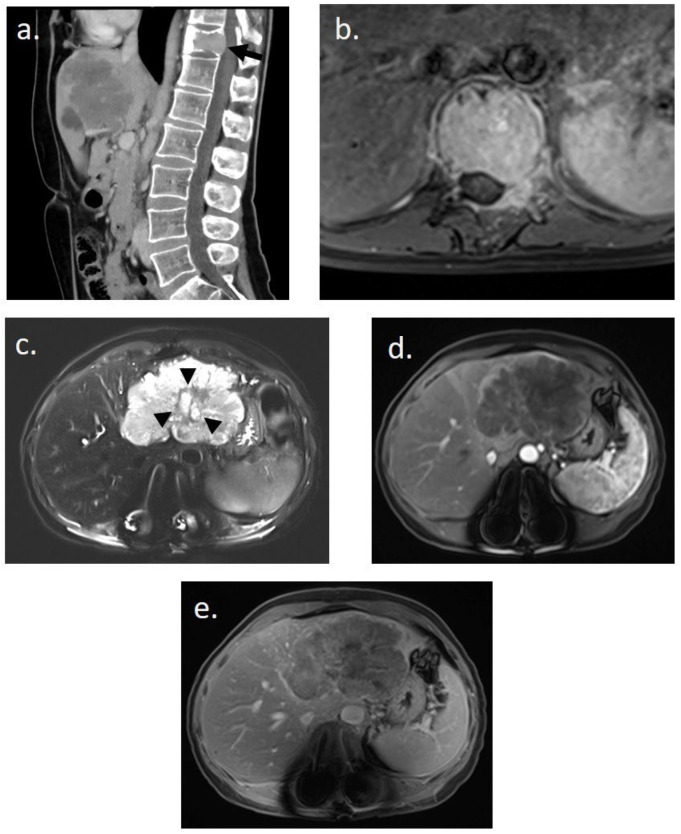
Initial imaging assessment for a 48-year-old woman presenting with lower back pain. (**a**) Abdomino-pelvic CT scan in sagittal slice post-contrast injection revealing a pathological fracture of T11 on a tissue-like lytic lesion (black arrow) and an infiltrating tissue lesion in the left hepatic lobe. (**b**) Spinal MRI in axial T1-weighted post-contrast injection showing complete tissue infiltration of the T11 vertebral body and the left posterior arch with epidural invasion. (**c**–**e**) Hepatic MRI performed immediately after spinal stabilization surgery. (**c**) Axial T2-weighted image with fat suppression showing a hyperintense polylobed lesion in the left lobe with a central possibly necrotic liquid zone (black arrowhead) on non-dysmorphic liver. (**d**,**e**) Enhanced axial T1-weighted images with fat suppression at (**d**) arterial and (**e**) 5 min delayed phases reveal a unique infiltrative hypovascular lesion with early peripheral rim enhancement. This imaging assessment was suggestive of a primary malignant hepatic lesion.

She underwent thoracic–abdominal–pelvic CT scan and spinal MRI on 20 March 2023 (Figure 1). Imaging revealed a 9 cm mass in the left liver, hypodense at different stages of contrast enhancement, with a central artery, dilatation of the intrahepatic bile ducts of segment II, and thrombosis of the left portal branch. There was no hepatic dysmorphism. Imaging also revealed an osteolytic lesion of the vertebral body of T11, with bulging of the posterior wall, canal narrowing, partial extension to the left pedicle, fracture with wedge-shaped compression, and invasion of the T11–D12 foramen on the left. The results suggested in the first instance a primary liver tumor associated with a vertebral metastasis.

She was first hospitalized to undergo urgent neurosurgical management with laminectomy, arthrodesis, and arthrectomy. Preoperative biopsy revealed a well-differentiated, monotypic kappa plasmacytic proliferation. Serum protein electrophoresis revealed monoclonal IgG Kappa (0.7 g/dL). Bone marrow aspirate confirmed the absence of clonal plasma cells. PET-CT performed on 18 April 2023 revealed a unique hypermetabolic bone lesion of T11 (SUVmax 41), in addition to a large hypermetabolic mass of the liver (SUVmax 9) related to cholangiocarcinoma, allowing the diagnosis of solitary bone plasmacytoma.

She had a liver biopsy of the hepatic lesion, which revealed an adenocarcinoma with expression of cytokeratin 19, cytokeratin 7, N-cadherin, and high expression of carbonic anydrase IX. In immunohistochemistry analysis, there was no HER2 expression, nor mismatch repair deficiency. No targetable mutation or fusion abnormality was detected by DNA next-generation panel sequencing (BRAF wild-type and microsatellite stable status, no IDH1 mutation) and RNA panel sequencing (notably, no FGFR or NTRK fusion).

**Figure 2:**
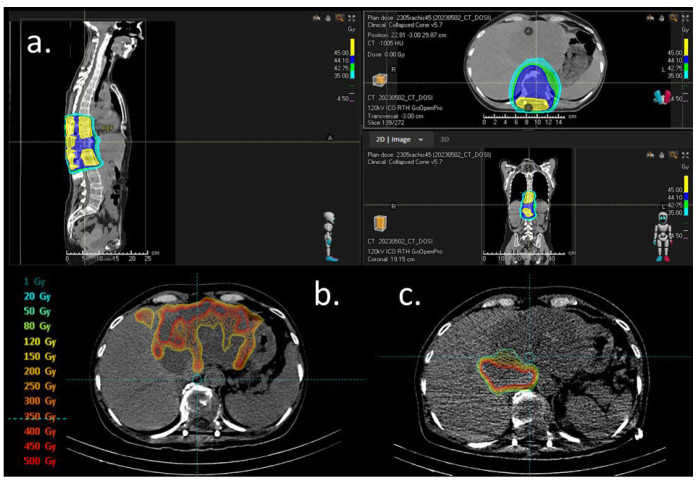
Dosimetry figures. (**a**) External beam radiation for solitary plasmocytoma. Isodose distribution in color wash, scale right. (**b**) Dosimetry simulation after 1st work-up with 99 mTc-macroaggregated albumin (MAA) injection in the left branch of the hepatic artery (23 May 2023). Distribution of the radiotracer on the left hepatic lesion with less uptake in two areas: segment II (initially attributed to a necrosis plage) and the second right side of the tumor (depending on another feeding artery). (**c**) Dosimetry simulation after second work-up with 99 mTc-MAA injection on a right artery branch (segment I and partially segment VIII on the CBCT) (8 June 2023). The distribution of the radiotracer showed less uptake than that in the first work-up.

Systemic therapy was initiated on 10 May 2023 with two cycles of capecitabine/cisplatin/durvalumab chemotherapy. The plasmacytoma was treated with IMRT to 45 Gy in 25 fractions from 17 May 2023 to 28 June 2023 (Figure 2a). In October 2023, imaging and biochemistry assessment of SP confirmed a complete response.

Selective internal radiation therapy (SIRT) with Yttrium-90 microspheres (Theraspheres^®^) was performed with Theraspheres^®^ injected into the left branch of the hepatic artery (23 May 2023) and during a second procedure in a branch of the right hepatic artery (8 June 2023, enabling targeting almost all of the lesion (a central plage with less uptake was initially attributed to the necrosis part of the lesion)). The total injected dose of Yttriu-m-90 was 7.5 GBq (Figure 2b,c).

**Figure 3:**
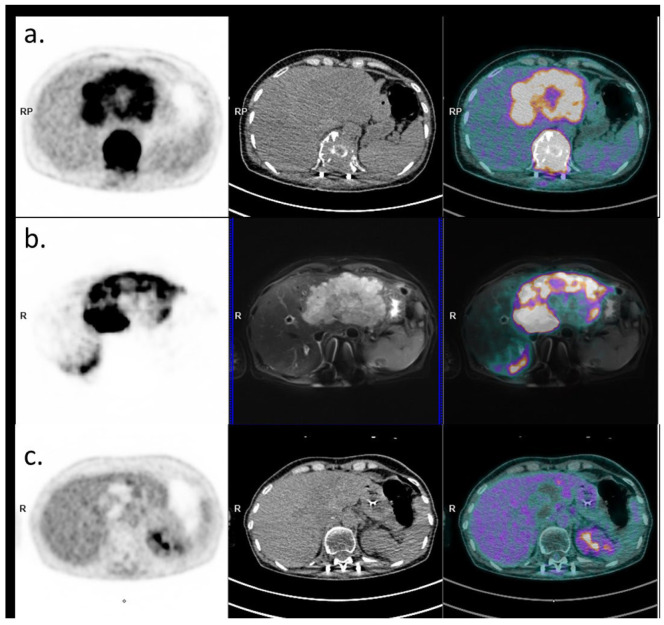
Baseline and post-therapeutic PET imaging (PET image on the left, CT on the middle, and fusion image on the right). (**a**) Initial 18FDG PET-CT evaluation (18 April 2024) showing the large hypermetabolic liver lesion and the hypermetabolic plasmacytoma. (**b**) PET-MRI evaluation post-Yttrium-90 injection with PET image on the left, MRI (axial T2 fatSat sequence) in the middle, and fusion on the right, showing good targeting of the liver lesion (22 June 2024). (**c**) 18FDG PET-CT evaluation 6 months later showing a complete metabolic response of the plasmacytoma and an excellent response of the liver lesion (persistence of a viable upper segment II residue).

Following both external radiation and SIRT, the chemotherapy regimen was switched to a GEMCIS durvalumab protocol for subsequent cycles. A CT scan in September 2023 showed RECIST stability and a partial mRECIST response in the cholangiocarcinoma. PET-CT performed in December 2023 revealed a partial metabolic response of the intrahepatic cholangiocarcinoma, and a complete metabolic response of the plasmocytoma (Figure 3). Durvalumab was continued as monotherapy and interrupted in May 2024 after one year of treatment. A surveillance program was then initiated.

## 3. Discussion

We report the case of a synchronous presentation of a hepatic lesion and a vertebral lesion, leading to the concomitant diagnosis of intrahepatic cholangiocarcinoma and vertebral plasmacytoma. Such a situation is a diagnostic trap, potentially leading to the conclusion of a metastatic lesion. A precise diagnosis and biopsy of two synchronous lesions should be discussed at a multidisciplinary meeting, to avoid misdiagnosing two different synchronous histological lesions, and to consider the treatment of both primary tumors rather than a metastatic disease. In the case of two primary tumors, one challenge is to identify the best therapeutic strategy, aiming at exploiting synergistic therapeutic effects and limiting cumulative toxicities. In the present case, radiation therapy has been used both for the treatment of plasmacytoma (external beam radiation therapy) and cholangiocarcinoma (intra-arterial radiation therapy). The durvalumab plus gemcitabine and cisplatin protocol was modified at the time of radiotherapy (SIRT and external radiotherapy) by replacing gemcitabine with capecitabine, to avoid the potential toxicity of the combination of gemcitabine and irradiation [1]. Both irradiations were concomitant with durvalumab.

There is growing evidence that the combination of radiotherapy and immunotherapy could have a synergistic effect. Immunity has been identified as a key factor in the response to radiotherapy. A dead tumor cell releases a cascade of signals and ligands into the microenvironment and expresses surface receptors that activate immunity. This process then results in the release of cytokines, cell death and damage factors, leading to interactions with T cells and dendritic cells [2]. Enhanced immunogenicity can be achieved with anti-CTLA-4, anti-PD-1, and anti-PD-L1 monoclonal antibodies [3,4]. Combining radiotherapy with an immunomodulator can induce a distant effect on non-irradiated sites, which is called the abscopal effect, highlighting the potential synergy [5]. Immunity has been identified as a key factor in the response to radiotherapy. A dead tumor cell releases a cascade of signals and ligands into the microenvironment and expresses surface receptors that activate immunity. This process then results in the release of cytokines, cell death and damage factors, leading to interactions with dendritic cells and T cells [2]. Anti-CTLA-4, anti-PD-1, and anti-PD-L1 monoclonal antibodies can potentiate tumor immunogenicity [3,4]. Radiation can also enhance the immune response by upregulating MHC class I, activating dendritic cells, increasing cross-presentation of tumor antigens, and promoting immune cell infiltration [2,5,6].

Biliary tract cancers may be classified into immune “hot” and “cold” depending on their cytotoxic lymphocyte (CTL) density. The immune “hot” type has a high density of CTLs and is associated with higher response rates to ICB, and vice versa [7]. In a retrospective analysis of surgical specimens of patients treated for HCC, without preoperative treatment (n = 32), after preoperative TACE (n = 16), or after preoperative SIRT (n = 2), SIRT was associated with a significant increase in Tumor-Infiltrating Lymphocytes, CD4+ and CD8+ T cells, and Granzyme-B expression compared to TACE or no preoperative treatment [8]. The combination of SIRT with gemcitabine and cisplatin demonstrated promising results in a prospective phase II study [9,10]. A recently published analysis derived from prospective clinical trials suggests that SIRT combined with chemotherapy might also improve outcomes over chemotherapy alone in patients with advanced liver-only iCCA [11]. The first-line treatment for advanced cholangiocarcinoma is now the combination of chemotherapy (gemcitabine/cisplatin) plus immunotherapy (durvalumab or pembrolizumab), regardless of mutational status [12,13]. Thus, there is a rationale for prospective randomized trials investigating combinations of SIRT and systemic treatments including immunotherapy.

A solitary bone plasmacytoma is defined as a single lytic lesion caused by monoclonal plasma cell infiltration, with or without soft tissue extension. Solitary plasmacytomas are highly sensitive to radiation, with local control ranging between 79 and 91%, giving the possibility for radiation therapy to be a curative treatment [14]. Depending on tumor size and localization, the recommended dose ranges between 40 and 50 Gy [15]. Radiotherapy is the standard of care for the treatment of solitary bone plasmacytoma, and no systemic therapy has been approved or recommended to date for these patients [16,17]. Even if the PD1/PDL1 axis seems to be an interesting target because most multiple myeloma cells express PDL1, the use of immune checkpoint inhibitors (ICIs) in monotherapy did not provide any clinical benefit in these patients in previous reports [18]. To our knowledge, there is no published experience of anti-PD-1/PD-L1 immunotherapy in patients with solitary plasmacytoma and therefore no data to support a potential synergy between this class of drug with radiotherapy in this context.

## 4. Conclusions

This case highlights the complexity of managing synchronous plasmacytoma and cholangiocarcinoma, with a challenging diagnostic approach, and the need for multidisciplinary management to exploit potential synergistic therapeutic effects, particularly those of combining radiotherapy and immunotherapy.

## Data Availability

The data presented in this study are available on request from the corresponding author.
